# Recent Advances in the Catalytic Asymmetric Reactions of Oxaziridines

**DOI:** 10.3390/molecules23102656

**Published:** 2018-10-16

**Authors:** Qiao Ren, Wen Yang, Yunfei Lan, Xurong Qin, Youzhou He, Lujiang Yuan

**Affiliations:** 1 Key Laboratory of Luminescent and Real-Time Analytical Chemistry (Southwest University), Ministry of Education, College of Pharmaceutical Sciences, Southwest University, Chongqing 400715, China; y12w151314@email.swu.edu.cn (W.Y.); lanyunfei96@163.com (Y.L.); xrqin@swu.edu.cn (X.Q.); 2 College of Environment and Resources, Chongqing Technology and Business University, Chongqing 400067, China; heyouzhou2016@163.com

**Keywords:** oxaziridines, asymmetric catalysis, oxidation, amination, aminohydroxylation

## Abstract

Oxaziridines have emerged as powerful and elegant oxygen- and nitrogen-transfer agents for a broad array of nucleophiles, due to the remarkably high and tunable reactivities. However, the asymmetric catalysis involving oxaziridines is still in its infancy. Herein, this review aims to examine recent advances in the catalytic asymmetric transformations of oxaziridines, including oxidation, amination, cycloaddition and deracemization.

## 1. Introduction

Oxaziridines, discovered in the mid-fifties by Emmons, are electrophilic three-membered heterocycles containing oxygen, nitrogen and carbon atoms [1]. Due to their strained three-membered ring and the virtue of their relatively weak N-O bond, they exhibit unusually high and tunable reactivities and have received considerable attention from chemists. Since their discovery, oxaziridines have emerged as important and powerful oxygen- and nitrogen-transfer reagents for a broad array of nucleophiles, including organometallic compounds [2], enolates, silyl enol ethers, alkenes, arenes, thiols, thioethers and selenides, nitrogen nucleophiles and C-H bonds in organic synthesis [3,4,5,6,7,8,9,10]. The reactive site of the nucleophilic attack on the oxaziridine heterocyclic ring is attributed to the electronic nature and size of the substituent on the 2-nitrogen atom. The introduction of an electron-withdrawing group on the 2-nitrogen atom, such as *N*-phosphinoyl and *N*-sulfonyl oxaziridines commonly known as Davis reagents, would increase the leaving ability of the nitrogen atom and, thus, facilitate the oxygen atom transfer. Furthermore, the larger size of the oxaziridine *N*-substituent generally results in higher levels of oxidation versus amination [11]. On the contrary, oxaziridines could serve as the electrophilic nitrogen transfer reagents when the *N*-substituent is small, such as *N*-H, *N*-alkoxycabonyl and *N*-alkyl oxaziridines. Additionally, they could also undergo some attractive and intriguing [2,3]-sigmatropic rearrangements [12,13,14,15], ring expansion [16], ring-opening process [17], desulfurization [18], C-H ethoxycarbonylation [19] and cycloaddition reactions, involving the N-O, C-C or C-O bond cleavage.

Moreover, the optically active and synthetically accessible oxaziridines [20,21] have emerged as crucial chiral building blocks in natural products and versatile enantiopure organic substrates in several enantioselective transformations, such as oxidation and amination, as well as some intriguing cycloaddition reactions with a large number of alkenes or alkynes to generate a diverse range of five-membered ring heterocycles. High yields and enantioselectivities were successfully achieved using the stoichiometric amount of enantiopure oxaziridines. Up to now, however, the catalytic versions of these transformations are still less reported. Thus, the present review covers the catalytic asymmetric reactions of oxaziridines and focuses on the synthetic applications rather than the detailed mechanistic pathways. The substrate-controlled and reagent-controlled asymmetric protocols are not pertinent for the present review.

## 2. Asymmetric Oxidation of Oxaziridines

**Figure 1:**
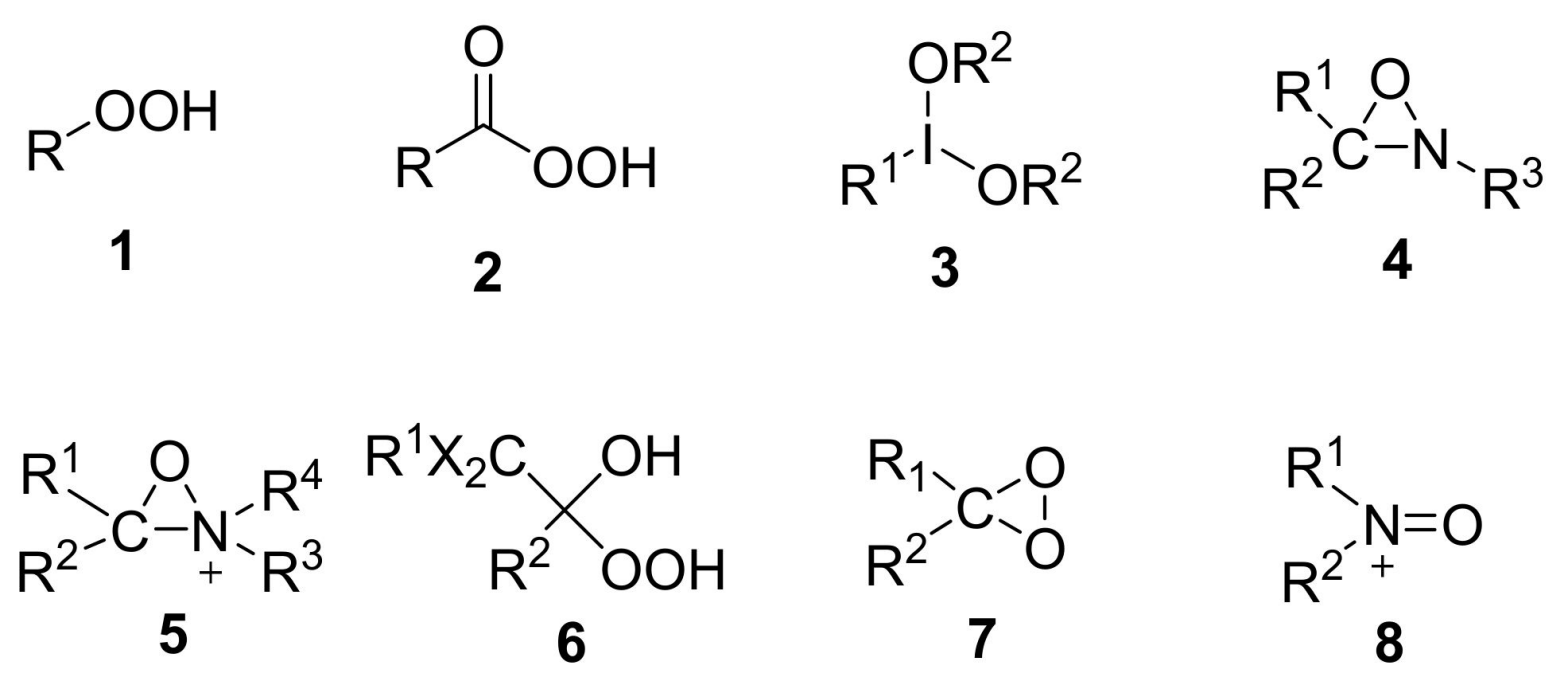
Representative nonmetal organic oxidants.

Due to the environmental benign, relative stability and operational simplicity, various nonmetal organic oxidants have been extensively employed in a broad range of oxidation transformations, most prominently hydroperoxides **1** (CHP, TBHP, H_2_O_2_), peroxy acid **2**, hypervalent iodine reagents **3** (PIDA, PIFA), oxaziridine **4**, oxaziridinium salt **5**, perhydrates **6**, dioxiranes **7** and oxoammonium salts **8** (Figure 1).

**Figure 2:**
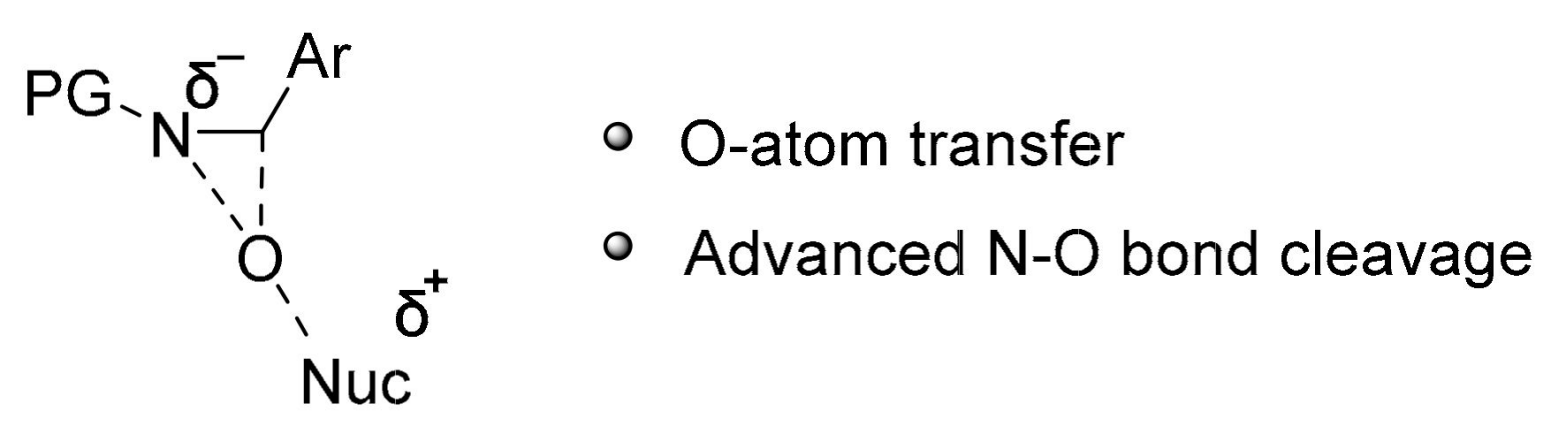
Asynchronous transition state for oxygen atom transfer reaction [10].

Remarkably, oxaziridine **4** has served as an important family of organic active oxidizing agents due to its unique oxygen transfer capability. A series of nucleophiles that include sulfides [22], sulfoxides, alkenes [23], thiolates [24], phenols [25,26], naphthols, enolates, silyl enol ethers, selenides [27] and C-H bonds [28,29,30,31] could be oxidized via an asynchronous transition state in which N-O bond cleavage is faster than C-O bond cleavage (Figure 2) [10]. Oxaziridinium salt **5**, first discovered by Lusinchi and co-workers in 1976 [32,33], was generated by the oxidation of the corresponding iminium salt with peracid or monoperoxysulfate. It exhibits a special oxidizing power derived from the strongly electrophilic oxygen atom. Moreover, the positive charge on the nitrogen atom significantly enhances the oxygen-transfer ability. Hence, this section will reveal the synthetic utility of oxaziridine **4** and oxaziridium salt **5** in oxidation stereochemistry.

### 2.1. Olefin Epoxidation

**Scheme 1:**
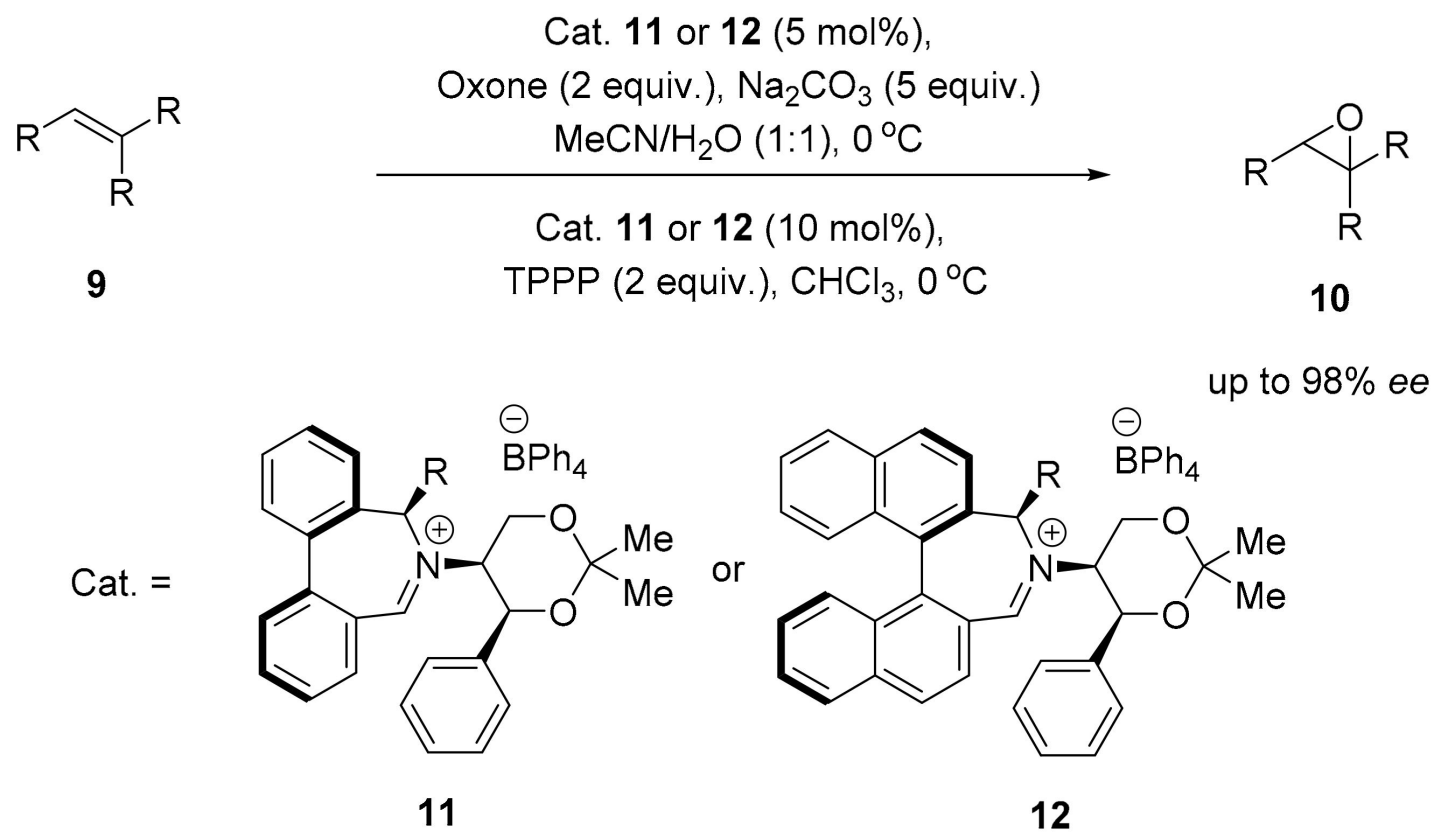
Asymmetric epoxidation of alkenes mediated by biphenyl or binaphthyl catalysts.

Consistent with the electron-deficient oxaziridine **4**, the positively charged oxaziridium salt **5** acts as the more powerful oxygen atom-transfer reagent rather than the nitrogen-transfer agent. Thus, the quaternized oxaziridinium salts could efficiently epoxidize alkenes at ambient temperature. They could be catalytically synthesized in situ from the corresponding iminium salts in the presence of a stoichiometric or excess oxidants. Page and co-workers employed iminium salt organocatalysts **11**/**12** in the asymmetric epoxidation of unfunctionalized alkenes **9** to generate optically active epoxides **10** (Scheme 1) [34]. Notably, the pseudoaxial substituent at a chiral center adjacent to the positively charged nitrogen atom in the binaphthyl- and biphenyl-derived salts significantly improved yields and enantioselectivities in many cases.

**Scheme 2:**
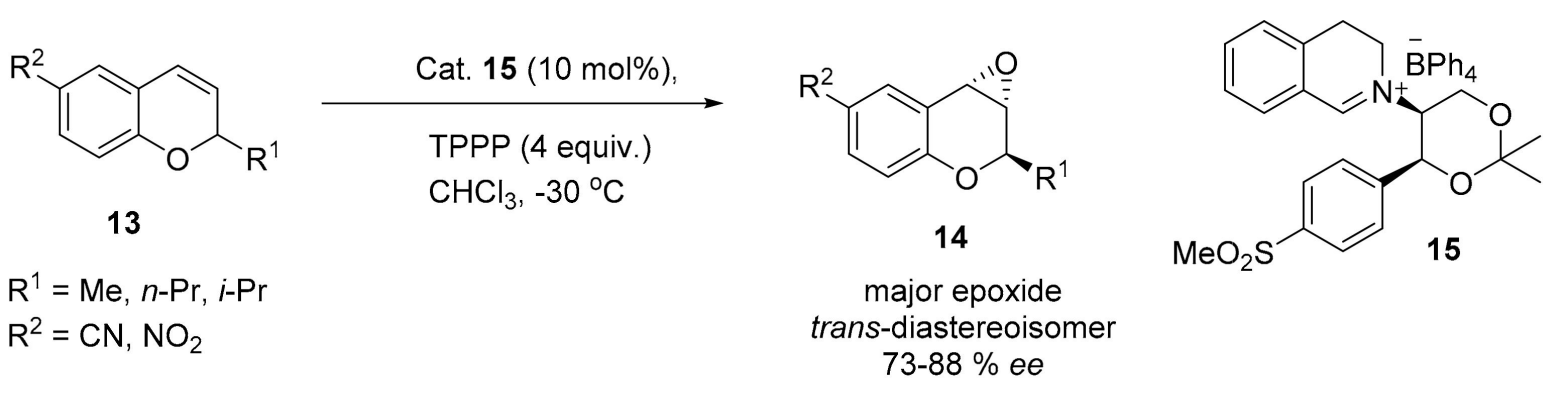
Kinetic resolution of 2-substituted chromene substrates.

Subsequently, they utilized the iminium salt **15**-catalyzed asymmetric epoxidation as a tool in the kinetic resolution of racemic 2-substituted chromene substrates **13**. Good enantioselectivities were achieved in all cases for the major epoxide diastereoisomers **14**, and the corresponding minor diastereoisomers revealed generally higher enantioselectivity (Scheme 2) [35]. The diastereoselectivity and enantioselectivity were partially dependent on the size of the substituent at C2 of the chromene scaffold.

### 2.2. Sulfoxidation

Enantiopure sulfoxides have emerged as very important chiral synthons and auxiliaries in asymmetric synthesis. One of the most convenient and straightforward protocols to generate the chiral sulfoxides is the asymmetric oxidation of sulfides. To date, the most efficient and successful protocols for the asymmetric sulfoxidation include the modified Sharpless procedures and oxidation using the stoichiometric quantity of enantiomerically pure Davis reagents or oxaziridinium salts [4,36,37,38]. However, asymmetric catalytic sulfoxidation, by comparison, has been less explored.

**Scheme 3:**
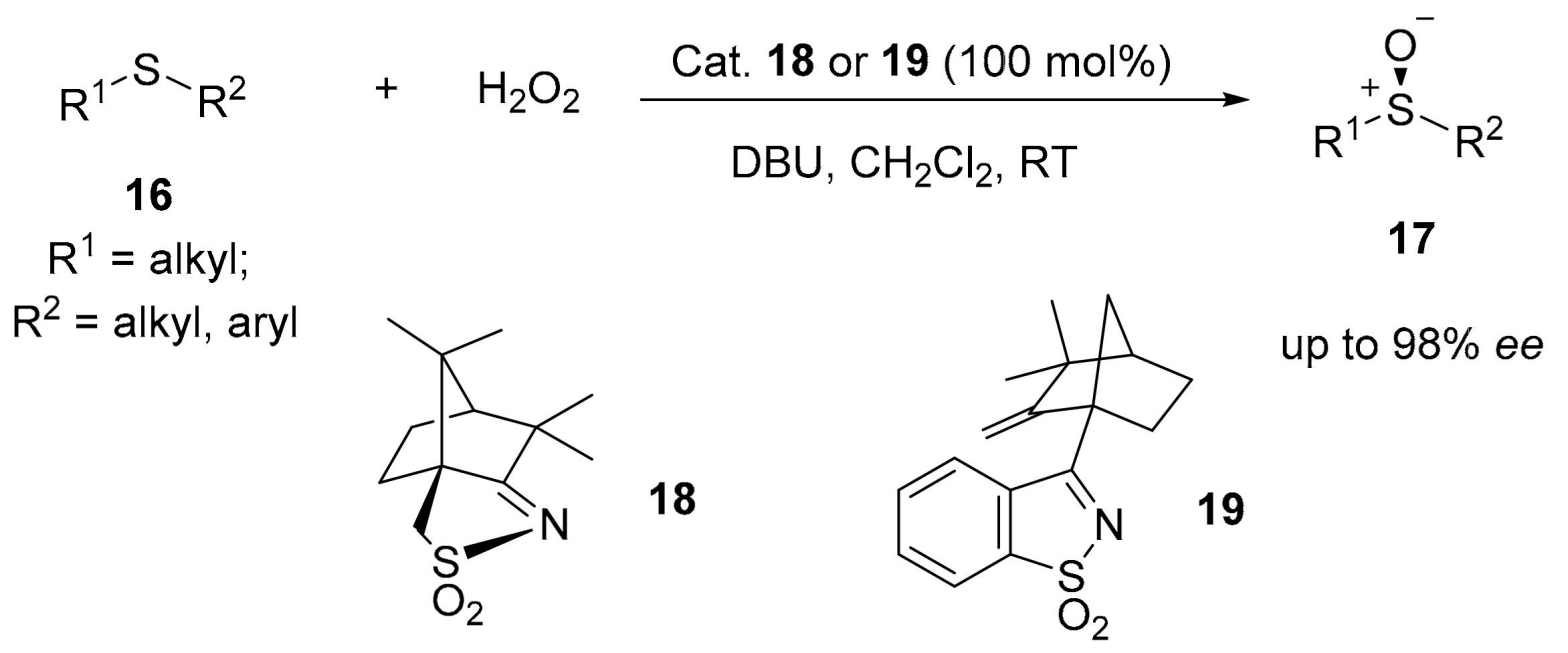
Enantioselective oxidation of sulfides.

In 1995, Page and co-workers demonstrated the initial research work on the catalytic asymmetric sulfoxidation. The [(3,3-dimethoxycamphoryl)sulfonyl]imine **18**, the precursor of the Davis reagent, was subjected to the terminal oxidant hydrogen peroxide to afford the chiral oxidant in situ, which promoted the asymmetric sulfoxidation of dialkyl sulfides **16** with excellent enantioselectivities (up to >98% *ee*) (Scheme 3) [39]. Five years later, the catalytic asymmetric oxidation of sulfides to sulfoxides was accomplished by the same group in the presence of the chiral nonracemic 3-substituted-1,2-benzisothiazole 1,1-dioxide **19** [40]. The stereochemical induction is determined largely by the absolute configuration of the carbon atom adjacent to the oxygen atom in the oxaziridine and α-hydroperoxyamine intermediates.

**Scheme 4:**
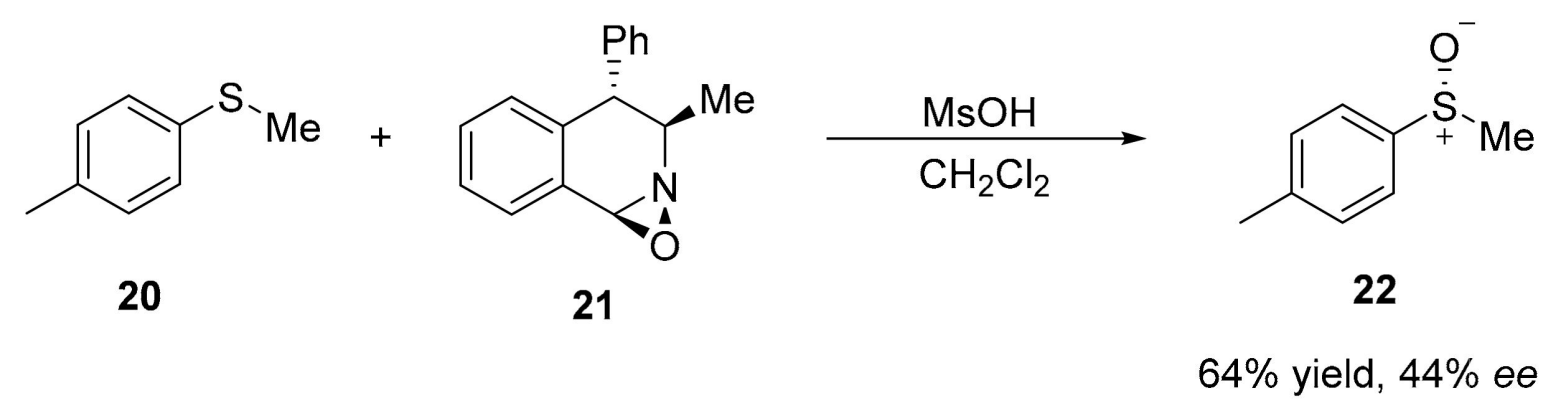
Acid-promoted asymmetric oxidation of sulfides to sulfoxides.

In comparison with the Davis reagents, normal *N*-alkyloxaziridines are less reactive in the oxygen transfer process. Thus, the introduction of exogenous additives has also been investigated in the asymmetric oxidation of sulfides. Bohé et al. employed the exogenous methanesulfonic acid (MsOH) to promote the asymmetric oxidation of sulfides **20** with chiral *N*-alkyl oxaziridine **21**, leading exclusively to the corresponding sulfoxides **22** (Scheme 4) [41]. Moreover, authors believed that the enhanced rate was attributed to the protonation of the basic nitrogen in the three-membered strained ring, affording the active oxaziridinium-like intermediates in situ.

**Scheme 5:**
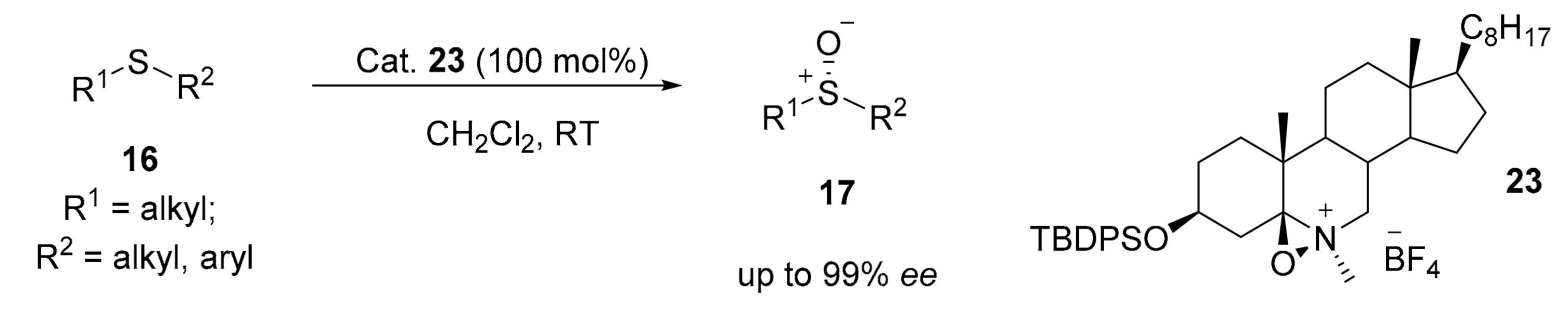
Sulfoxidations with oxaziridinium **23**.

**Scheme 6:**
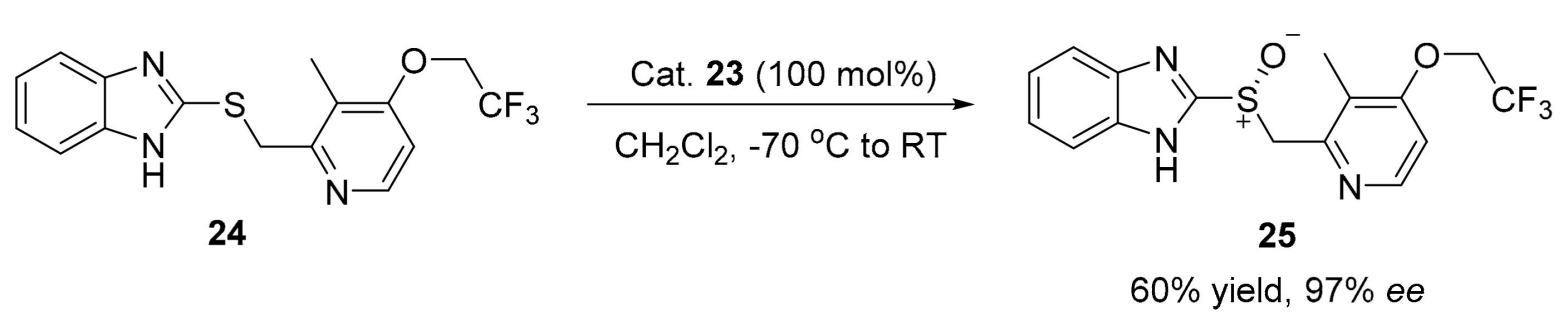
Asymmetric synthesis of (*R*)-lansoprazole **25**.

Therefore, a series of chiral oxaziridinium salts were developed to promote the asymmetric oxidation of sulfides to sulfoxides. In 2007, Bohé group synthesized a novel and effective oxaziridinium salt **23** for the highly enantioselective sulfoxidation in good yields and with up to >99% *ee* (Scheme 5) [42]. The synthetic utility of this protocol was demonstrated by the asymmetric synthesis of the biologically active proton pump inhibitor (*R*)-lansoprazole **25**, a well-known sulfinyl-substituted benzimidazole, from the oxidation of sulfide **24** by oxaziridinium salt **23** (Scheme 6). The excellent enantioselectivity (97% *ee*) and good yield (60%) were obtained in this transformation.

### 2.3. Enolate Oxidation

The hydroxylation of an enolate is one of the most practical and efficient protocols for the introduction of a hydroxyl group adjacent to a carbonyl group [3]. Since the pioneering work by the Davis group in the 1980s [43,44,45], *N*-sulfonyloxaziridines have served as the preferred oxidizing reagents in this transformation, due to the high efficiency, commercial availability, lack of byproducts and ease of work-up. Nevertheless, the asymmetric oxidation reaction of enolates [46,47,48,49,50], *aza*-enolates [51] and phosphonate anions [52] generally require a stoichiometric amount of chiral oxaziridine or auxiliary.

**Scheme 7:**
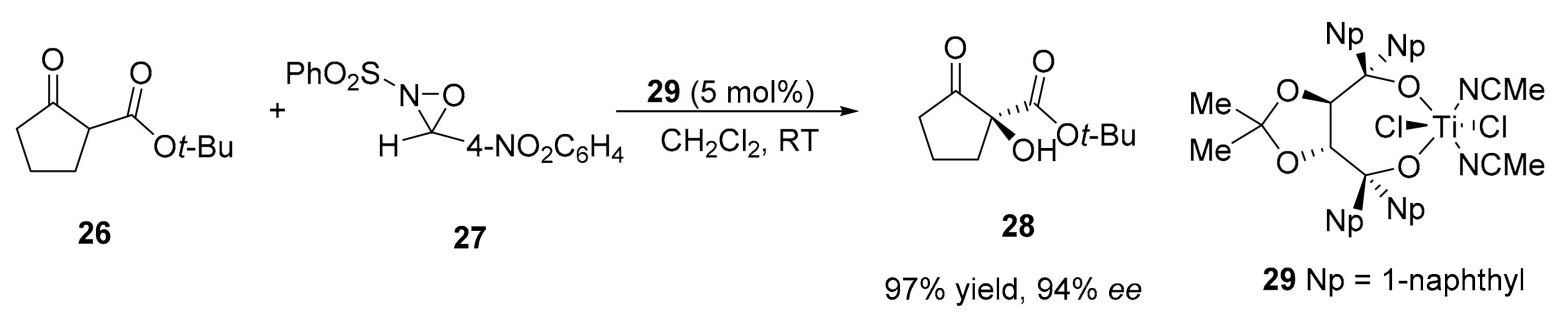
Titanium-catalyzed asymmetric hydroxylations.

Togni and co-workers reported an initial research concerning the catalytic enantioselective α-hydroxylation of various β-keto esters **26** catalyzed by a chiral titanium complex [TiCl_2_((*R,R*)-1-Np-TADDOLato)(MeCN)_2_] **29** (Scheme 7) [53]. A variety of α-hydroxylated products **28** were obtained in high yields and enantioselectivities (up to 94% *ee*) with racemic *N*-sulfonyloxaziridine **27** as the terminal oxidizing agent. However, the enantioselectivity relied on the size of the ester substituent. An ester substituent smaller than the *tert*-butyl group enabled the modest enantioinduction in this transformation. Moreover, the authors proposed a possible mechanism involving the formation of chiral titanium-bound enolate via the coordination of the Lewis acid complex **29** to the β-keto ester substrate **26**, epoxidation of the enolate and subsequent ring-opening process to the final 2-hydroxylated 1,3-dicarbonyl compound **28**. A few years later, an asymmetric Cu(I)-, Pd(II), Zn(II) or Fe(III)-catalyzed α-hydroxylation of β-keto esters has also been reported by Shi [54,55], Hii [56], Ding [57] and Che [58] group, respectively.

**Scheme 8:**
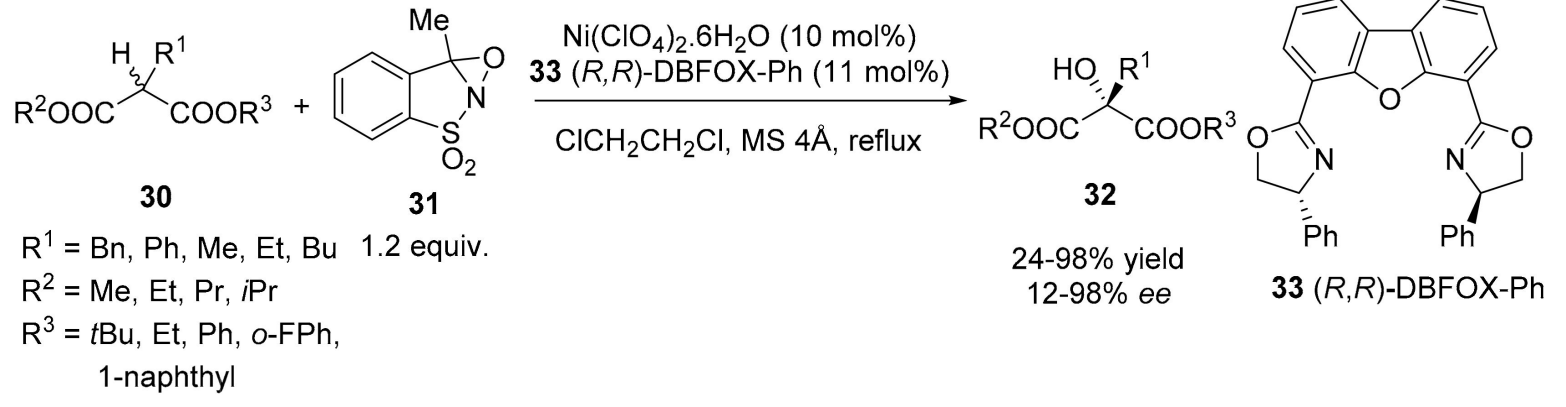
Catalytic enantioselective hydroxylation of racemic malonates **30**.

In 2009, the first dynamic kinetic enantioselective α-hydroxylation of racemic malonates **30** and oxaziridine **31** was achieved by the Shibata group [59]. The (*R*,*R*)-DBFOX-Ph **33**/Ni^II^ complex was employed to promote this transformation to generate the chiral α-hydroxy malonate **32** with a quaternary stereocenter in high yield and enantioselectivity (up to 98% *ee*) (Scheme 8).

**Scheme 9:**
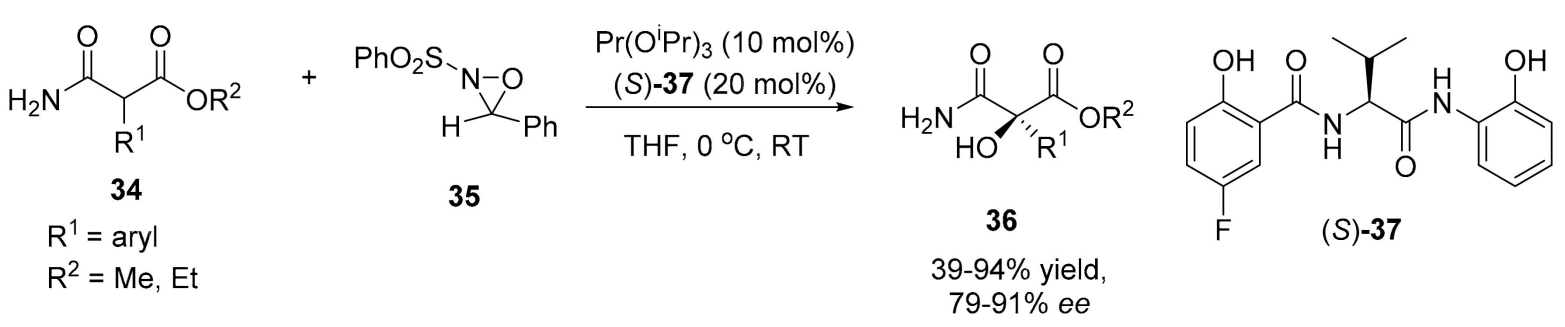
Catalytic asymmetric hydroxylation of *N*-nonsubstituted α-alkoxycarbonyl amide.

Furthermore, the less active pronucleophile *N*-nonsubstituted α-alkoxycarbonyl amide **34** with the higher p*K*a of α-hydrogen than the related 1,3-dicarbonyl compound was oxidized via a catalytic asymmetric hydroxylation (Scheme 9) [60]. Shibasaki et al. established the prasedymium isopropoxide [Pr(O*i*Pr)_3_] and a fluoro-substituted amide-based-ligand (*S*)-**37** as the optimal and effective catalytic system and *N*-sulfonyl oxaziridine **35** as the oxidizing reagent. The authors believed the praseodymium salt formed a complex with *N*-nonsubstituted α-alkoxycarbonyl amide **34**, whereas, the amide-based ligand (*S*)-**37** and oxaziridine **35** assembled together through the coordination and hydrogen bonding resulting in the associated transition state. Thus, the *trans* amide N-H proton is particularly crucial to the assembled transition state.

**Scheme 10:**
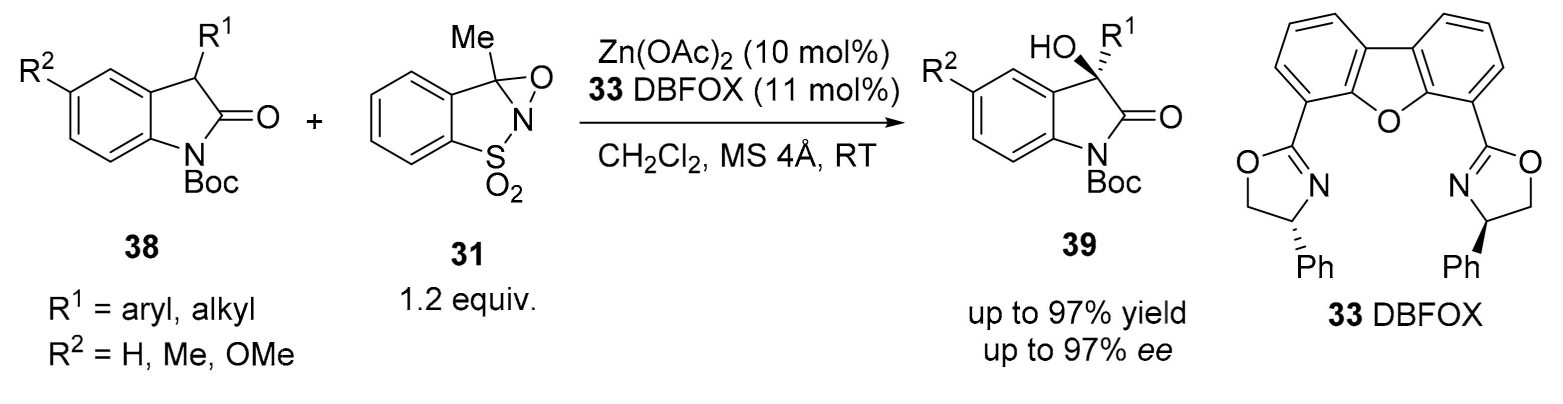
Enantioselective hydroxylation of 3-alkyl and 3-aryl-2-oxindoles.

The Shibata group reported the pioneering work on the zinc (II)-catalyzed asymmetric catalytic hydroxylation reaction of both 3-alkyl and 3-aryl-2-oxindoles **38** (Scheme 10) [61]. This protocol utilized the chiral DBFOX-Zn(II) complex as the catalyst and racemic saccharin-derived oxaziridine **31** as the terminal oxidant, leading to the enantioselective synthesis of the pharmaceutically important chiral 3-hydroxy-2-oxindoles **39** with up to 97% *ee*. Moreover, this methodology has been successfully applied to the enantioselective hydroxylation of β-keto esters. Subsequently, Naganawa and co-workers discovered an unprecedented Cu(II)-catalyzed enantioselective oxygen transfer reaction of 3-aryl-2-oxindoles with racemic Davis oxaziridine using the original phenanthroline as *N*,*N*,*O*-tridentate ligand [62]. More recently, a chiral iminophosphorane organocatalyzed enantioselective hydroxylation of 3-substituted oxindoles was developed with racemic oxaziridine, leading to the construction of optically active 3-substituted-3-hydroxy-2-oxindoles in excellent yields and moderate to excellent enantioselectivities [63]. 

**Scheme 11:**
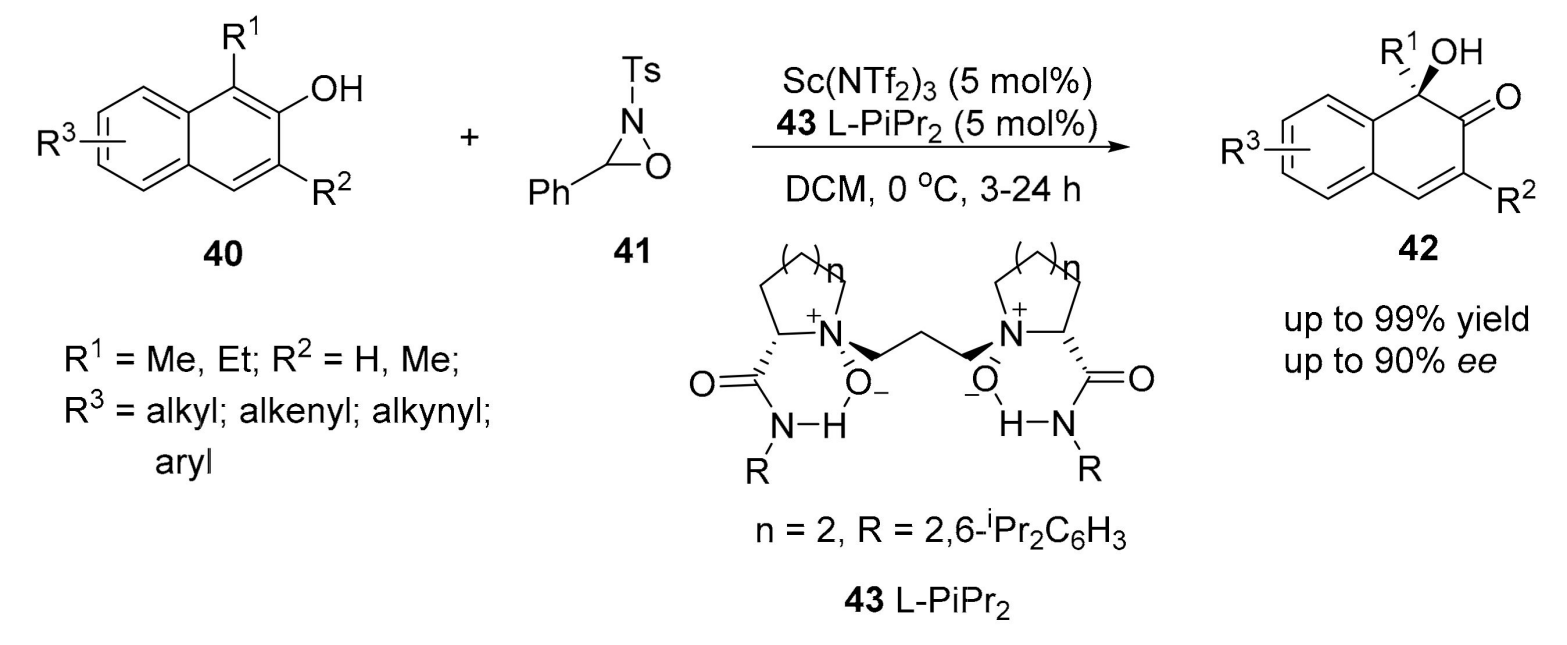
Catalytic asymmetric hydroxylative dearomatization of 2-naphthols.

Recently, a highly chemo- and enantioselective hydroxylative dearomatization of 2-naphthols **40** with racemic oxaziridines **41** was accomplished by the Feng group using a *N*,*N*′-dioxide-scandium(III) complex catalyst (Scheme 11) [64]. This approach afforded a number of substituted *ortho*-quinols **42** in high yields and enantioselectivities. Notably, the choice of the scandium salt’s counterion efficiently suppressed the α-ketol rearrangement. The desired *R*-configured product **42** was generated by the *Si*-face attack of the oxaziridine on the α-position of 2-naphthol. 

**Scheme 12:**
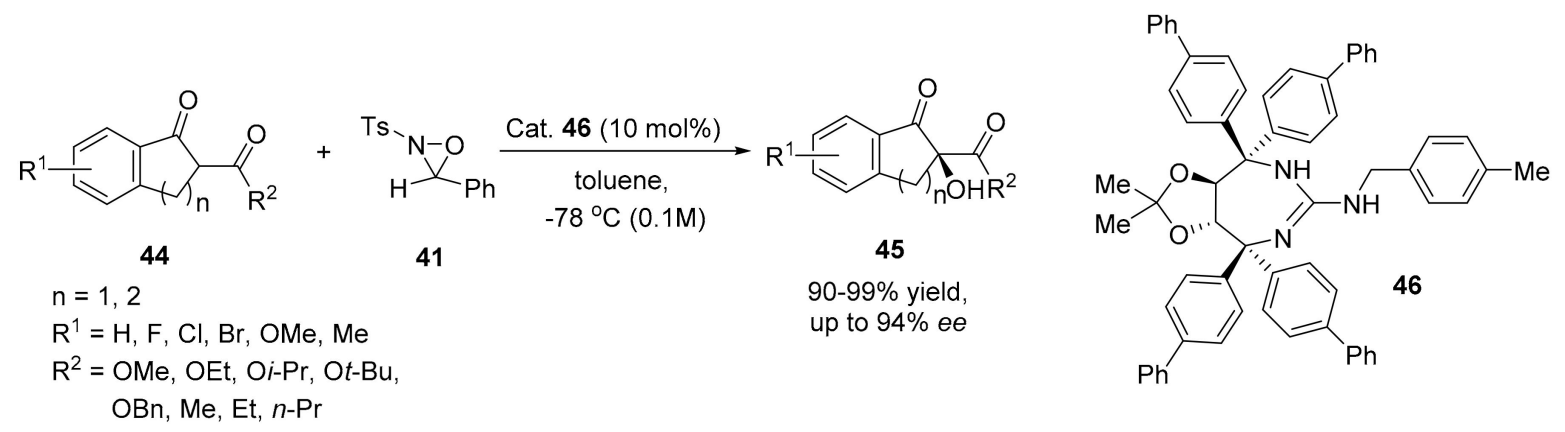
Chiral guanidine-catalyzed enantioselective α-hydroxylation.

In addition to the above-mentioned Lewis acid-mediated catalytic asymmetric α-hydroxylation, there have been some highly enantioselective organocatalytic protocols to α-hydroxy carbonyl compounds with oxaziridines as oxidants. Zou et al. disclosed the construction of a novel library of guanidines **46** derived from ethyl l-tartrate as the chiral source and their application to catalytic asymmetric α-hydroxylation of β-keto ester and β-dicarbonyl substrates **44** with remarkable efficiency and excellent enantioselectivity (Scheme 12) [65].

**Scheme 13:**
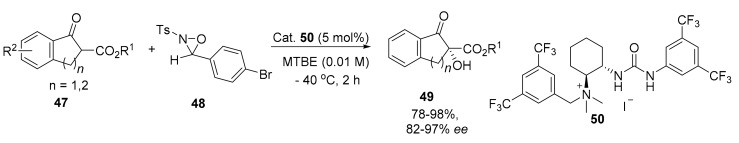
Asymmetric catalytic α-hydroxylation reactions of β-keto esters.

Additionally, chiral bifunctional urea-containing ammonium salt **50** was developed by the Waser group to be a very efficient catalyst for asymmetric catalytic α-hydroxylation reactions of various β-keto esters **47** with racemic oxaziridines **48** with good to excellent enantioselectivities (82–97% *ee*) (Scheme 13) [66]. Simultaneously, this process is also accompanied by a kinetic resolution of the oxaziridine **48**.

**Scheme 14:**
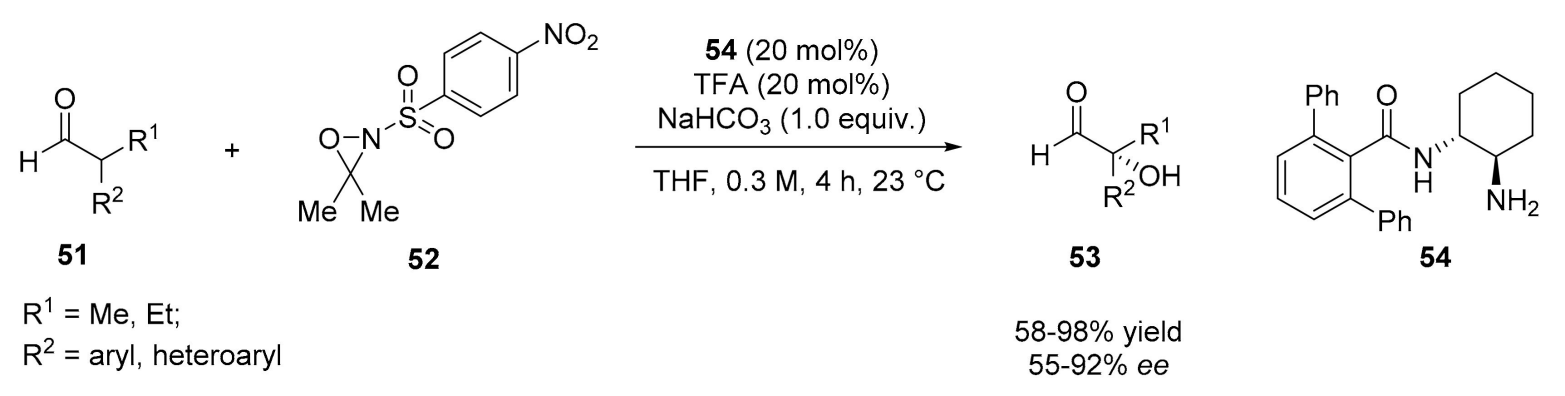
Enantioselective α-hydroxylations of branched aldehydes.

In 2015, the Jacobsen group described a novel and simple aminobenzamide catalyst **54** for the asymmetric α-hydroxylation of α,α-disubstituted aldehydes **51** with oxaziridine **52** developed by the Yoon group (Scheme 14) [67]. The α-hydroxy aldehydes bearing tetrasubstituted, stereogenic centers **53** were generated in excellent yields with high enantioselectivities. The stereoselectivities of these transformations mainly depended on the *E*/*Z* ratio of the key enamine intermediates. 

### 2.4. Rubottom Oxidation

**Scheme 15:**
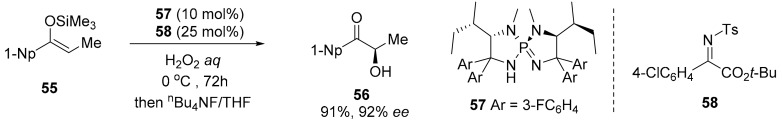
Asymmetric catalytic Rubottom oxidation.

More recently, the Ooi group synthesized a novel class of chiral *N*-sulfonyl oxaziridines as uniquely reactive chiral organic oxidants [68]. Notably, they developed an asymmetric catalytic Rubottom oxidation of silyl enol ether **55** catalyzed by the requisite chiral *N*-sulfonyl oxaziridine, in situ generated by the oxidation of a catalytic amount of the parent α-imino ester **58**, with L-isoleucine-derived triaminoiminophosphorane **57** as an organocatalyst and H_2_O_2_ as a stoichiometric terminal oxidant (Scheme 15).

## 3. Asymmetric Amination of Oxaziridines

Electrophilic amination is an important Nu-N bond formation reaction in which an electron-poor nitrogen is transferred to an electron-rich carbon [69,70,71,72], nitrogen [73,74,75], sulfur [76] or phosphorus nucleophile. Collet and co-workers completed a pioneering work in which aldehyde-derived *N*-Boc-oxaziridines could promote the electrophilic amination of various nucleophiles [11,77]. Notably, the *N*-substituent on the nitrogen plays a significant role in the oxaziridine reactivities. In fact, *N*-H, *N*-alkoxycabonyl and *N*-alkyl oxaziridines are usually utilized as electrophilic aminating agents, presumably due to the small substituent on the nitrogen atom.

However, the amination process is always hampered by the competitive oxidation and aldol reaction. Furthermore, oxaziridines could also be converted to amides via the transition metal-catalyzed radical rearrangement [78]. Thus, the combination of this rearrangement with the previous oxaziridine synthesis is a synthetically valuable process to generate amides from carbonyl compounds.

Additionally, asymmetric versions of electrophilic amination of oxaziridines generally require stoichiometric amounts of chiral reagents or the presence of enantiomeric induction of the substrates. In 1998, Enders et al. disclosed the nitrogen atom transfer reactivity of *N*-Boc oxaziridines in the asymmetric electrophilic amination [79]. The stereochemistry of chiral α’-silyl ketones substrates was transferred to the final desired α-amino ketones.

**Scheme 16:**
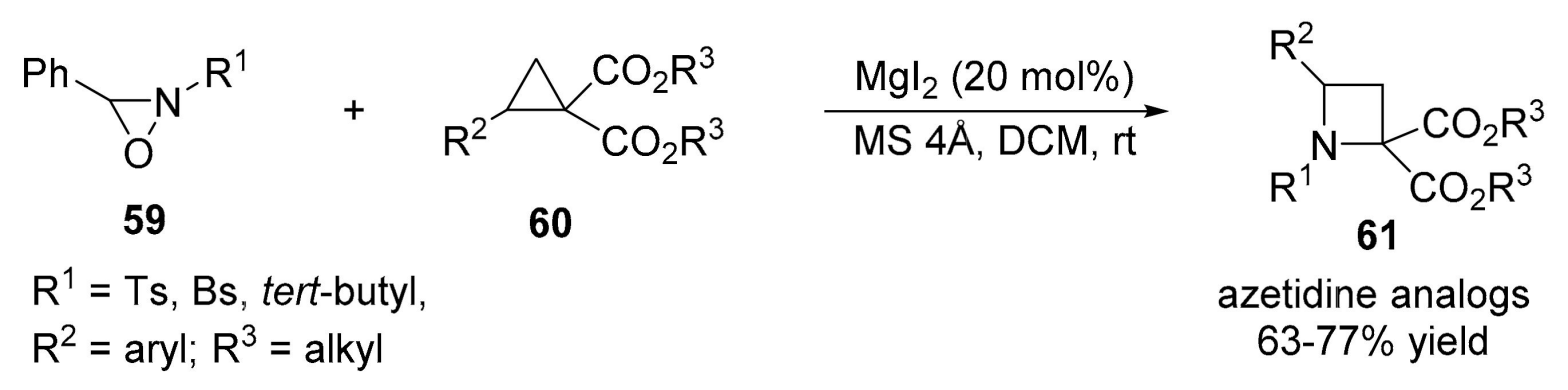
Ring expansion of donor-acceptor cyclopropane.

Recently, the Banerjee group demonstrated the unique *N*-substituent directed dual reactivity of oxaziridine **59** toward donor-acceptor cyclopropane **60** (DAC) (Scheme 16) [80]. The ring expansion of DAC was accomplished via the *N*-substituent controlled selective *N*-transfer of oxaziridines, directly leading to the azetidine derivatives **61** in moderate to good yields. Interestingly, the *N*-alkyl substituted oxaziridines with α-hydrogen like *N*-methyl, *N*-isopropyl led to the pyrrolidine derivatives through a [3+2] cycloaddition reaction between DACs and in situ generated imine.

## 4. Asymmetric Cycloaddition of Oxaziridines

In 1997, the Dmitrienko group accomplished an anomalous aminohydroxylation of 2-benzenesulfonyl-3-aryloxaziridines (Davis reagents) with indoles to yield the unusual 1,3-oxazolidinoindole rings rather than the indole 2,3-epoxides or their derivatives [81]. Subsequently, novel copper(II)-catalyzed aminohydroxylations of various styrenes and 1,3-dienes were demonstrated by the Yoon group to furnish 1,3-oxazolidines [82,83,84].

**Scheme 17:**
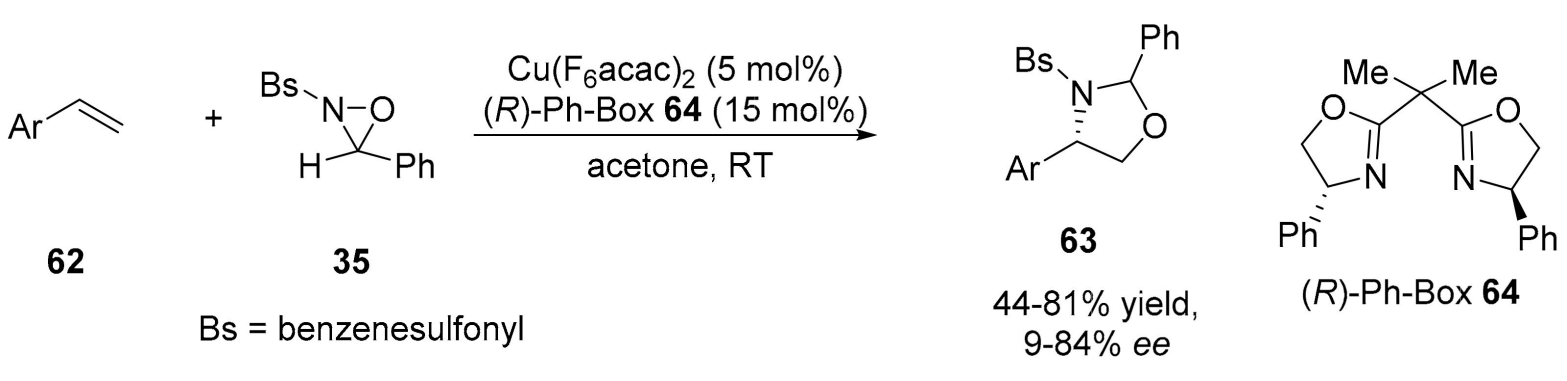
Oxaziridine-mediated enantioselective aminohydroxylation of styrenes **62**.

Additionally, Yoon and co-workers reported the initial works toward the asymmetric aminohydroxylation catalyzed by a chiral copper(II) bis(oxazoline) complex (Scheme 17) [85]. Good yields and modest to good enantioselectivities were achieved in the aminohydroxylation of a variety of styrenes **62** with racemic *N*-sulfonyl oxaziridine **35** (Davis oxaziridine) in the presence of commercially available copper(II) hexafluoroacetylacetonate [Cu(F_6_acac)_2_)] and chiral ligand (*R*,*R*)-Ph-Box **64**. Besides, the enantiopurities of the resulting amino alcohols upon acid-catalyzed hydrolysis of **63** could be improved to very high levels (>99% *ee*) through recrystallization. This protocol is a crucial complement to the enantioselective Sharpless aminohydroxylation reaction [86] and the regioselectivity is much higher.

**Scheme 18:**
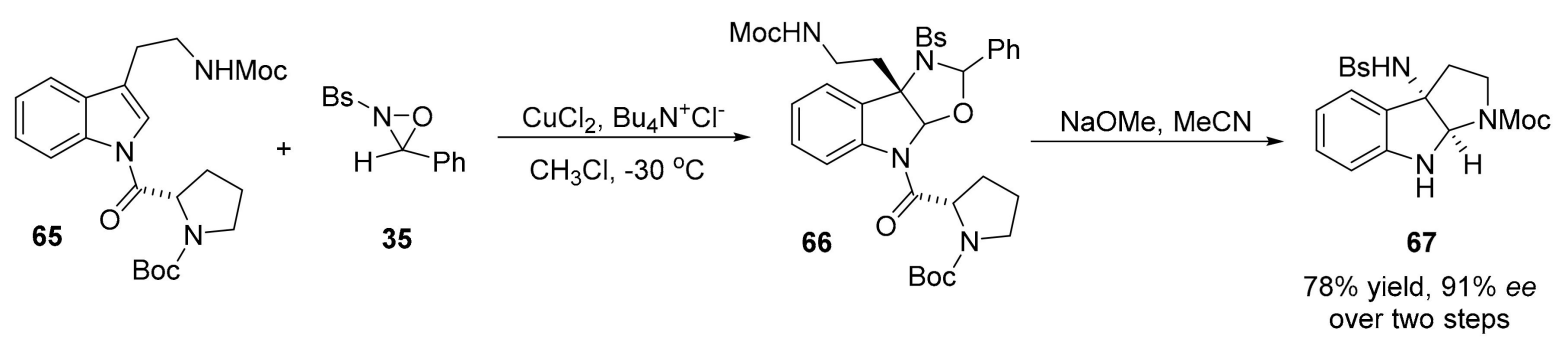
Synthesis of enantioenriched 3-aminopyrroloindolines **67**.

They quickly discovered, however, that anionic halocuprate(II) complexes CuCl_2_/Bu_4_N^+^Cl^−^ could serve as remarkably more active catalysts for aminohydroxylation of less reactive oxaziridines (3,3-dimethyl oxaziridines) than neutral copper(II) salts [87]. In addition, these halocuprate catalyst systems have been applied to the aminohydroxylation of *N*-acyl indole derivatives **65** with racemic *N*-sulfonyl oxaziridines **35**. When a chiral *N*-acyl group was applied, the resulting aminal products **66** could be converted in a single step into the enantiomerically enriched 3-aminopyrroloindoline **67**, a common structural core present in numerous biologically active indole alkaloids (Scheme 18) [88].

**Scheme 19:**
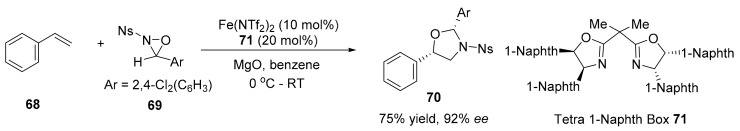
Iron catalyzed asymmetric aminohydroxylation of olefin **68**.

As a complement of the analogous copper(II)-catalyzed reaction, the Yoon group reported a highly enantioselective and regioselective aminohydroxylation catalyzed by a combination of a highly electron-deficient iron(II) triflimide and bis(oxazoline) ligand **71** (Scheme 19) [89]. The reaction of alkenes **68** and *N*-sulfonyl oxaziridine **69** afforded a variety of oxazolidine products **70**, which could be easily manipulated to construct highly enantioenriched free amino alcohols under the standard acid-catalyzed hydrolysis conditions. Thus, these oxaziridine-mediated asymmetric aminohydroxylations of olefins **68** could yield both regioisomers of optically active 1,2-aminoalcohols. Moreover, the regiochemistry was mainly dependent on the choice of transition metal catalyst.

**Scheme 20:**
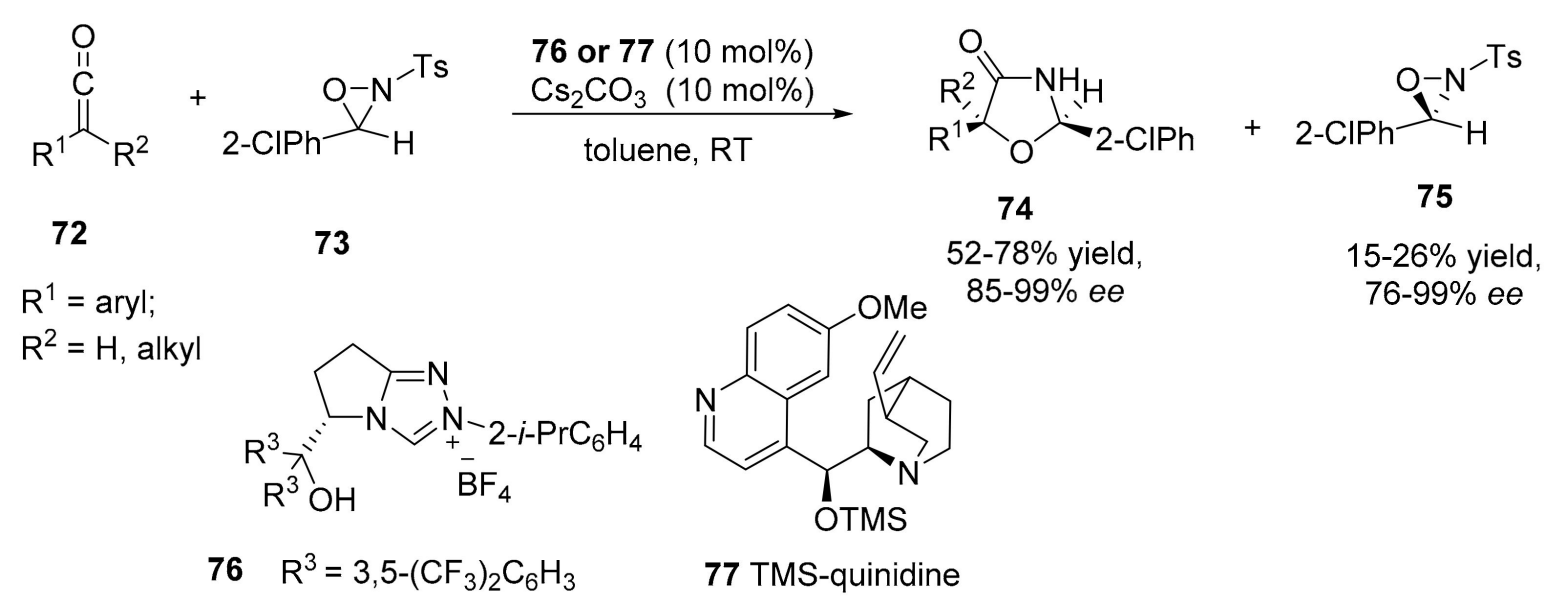
Formal [3+2] cycloaddition reaction of ketenes and oxaziridines.

**Scheme 21:**
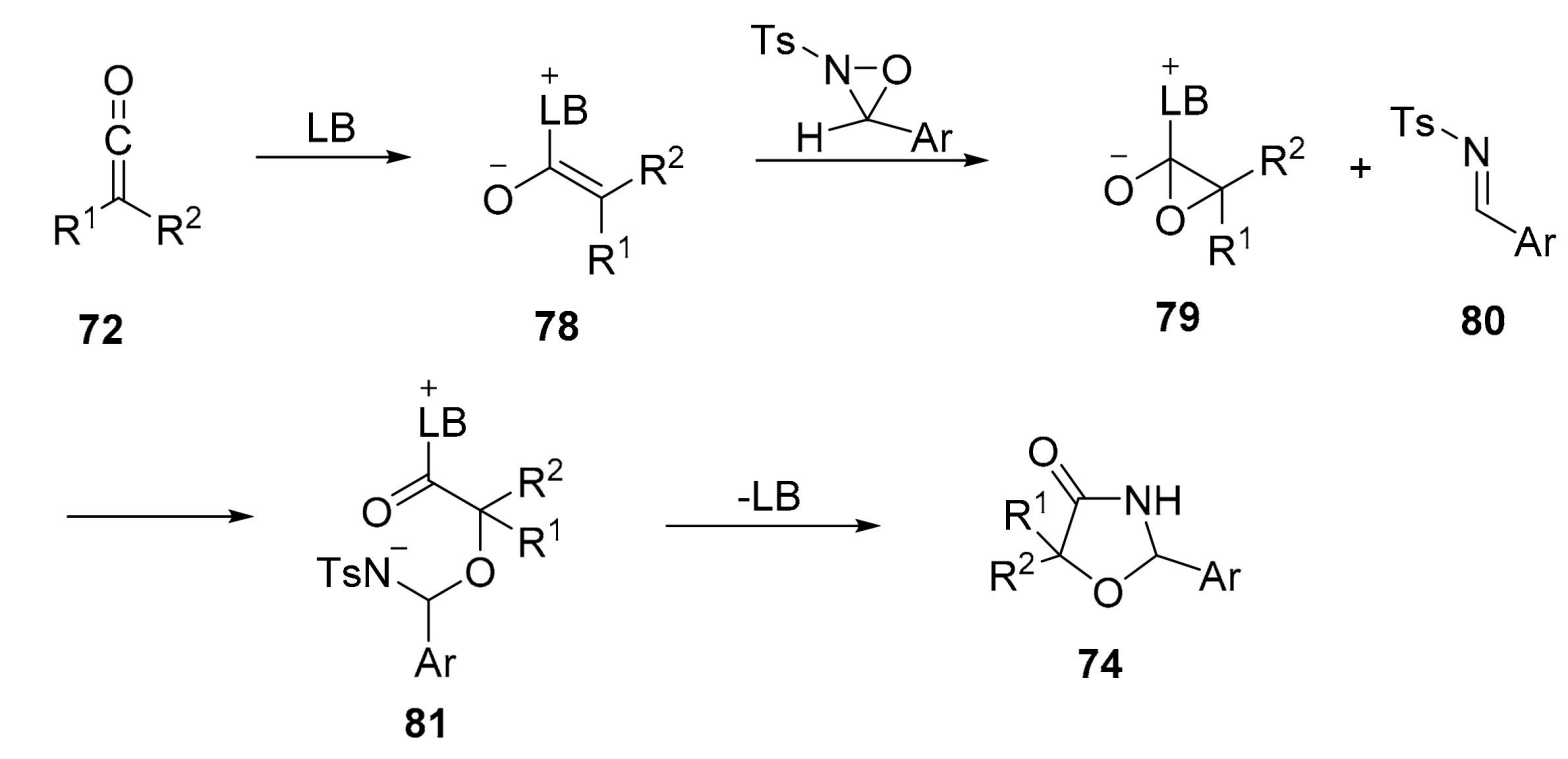
Mechanistic proposal for Lewis base-catalyzed cycloaddition.

Moreover, Ye and co-workers have demonstrated an organocatalytic enantioselective aminohydroxylation catalyzed by chiral Lewis bases (Scheme 20) [90]. This formal [3+2] cycloaddition employed ketenes **72** and oxaziridines **73** to generate a series of oxazolidin-4-ones **74** in moderate to good yields with good diastereo- and enantioselectivities via N-O bond cleavage. *N*-heterocyclic carbene **76** and cinchona alkaloid **77** acted as the optimal organocatalyst for the reaction of stable disubstituted ketenes and unstable monosubstituted ketenes, respectively. In addition, they believed that this transformation was initialized by the formation of the zwitterionic enolate intermediate **78**, which attacked the electrophilic oxygen of the oxaziridine **73** to afford the transient epoxide species **79** and the imine **80**. Subsequent addition of nucleophilic intermediate **79** with the imine **80** furnished the final cyclic product and regenerated the Lewis base catalyst (Scheme 21).

Recently, an asymmetric chiral *N*-heterocyclic carbene catalyzed formal [3+2] cycloaddition of α-aroylxoxyaldehydes and enantiopure oxaziridines was developed by the Smith group for the generation of oxazolidin-4-one products [91]. Excellent diastereo- and enantioselectivities were achieved. Acyl azolium enolate, in situ generated from the α-aroylxoxyaldehyde and NHC catalyst, attacked the electrophilic oxygen of chiral oxaziridine resulting in α-oxidation and N-O bond cleavage of the oxaziridine.

**Scheme 22:**
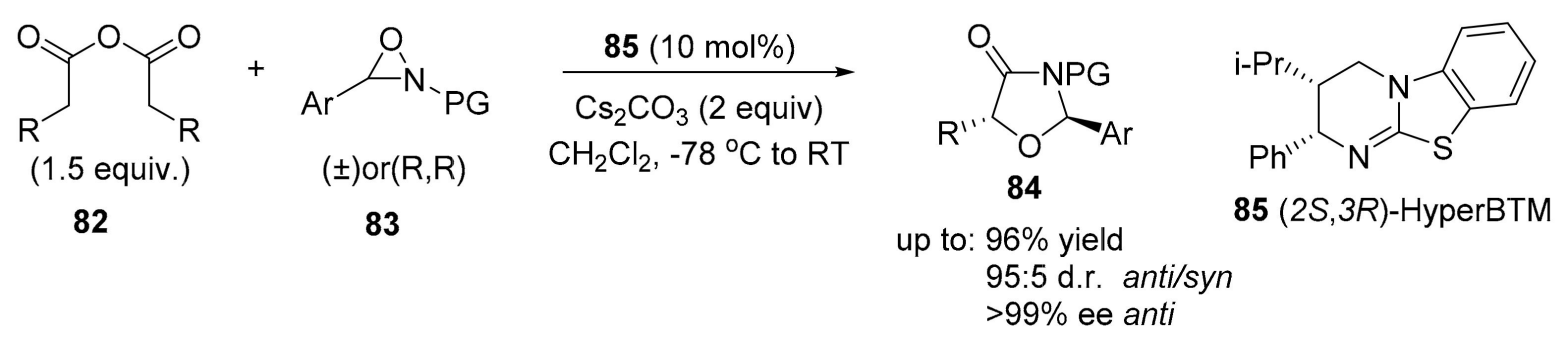
Formal [3+2] cycloaddition of ammonium enolates with oxaziridines.

Besides, the commercially available isothiourea catalyst (2*S*,3*R*)-HyperBTM **85** was utilized to promote the formal [3+2] cycloaddition of homoanhydrides **82** and oxaziridines **83** by the Smith group (Scheme 22) [92]. This process allowed the assembly of stereodefined oxazolidin-4-ones **84**, which could be subjected to the deprotection and reduction for the generation of enantioenriched α-hydroxy carboxylic acid.

**Scheme 23:**
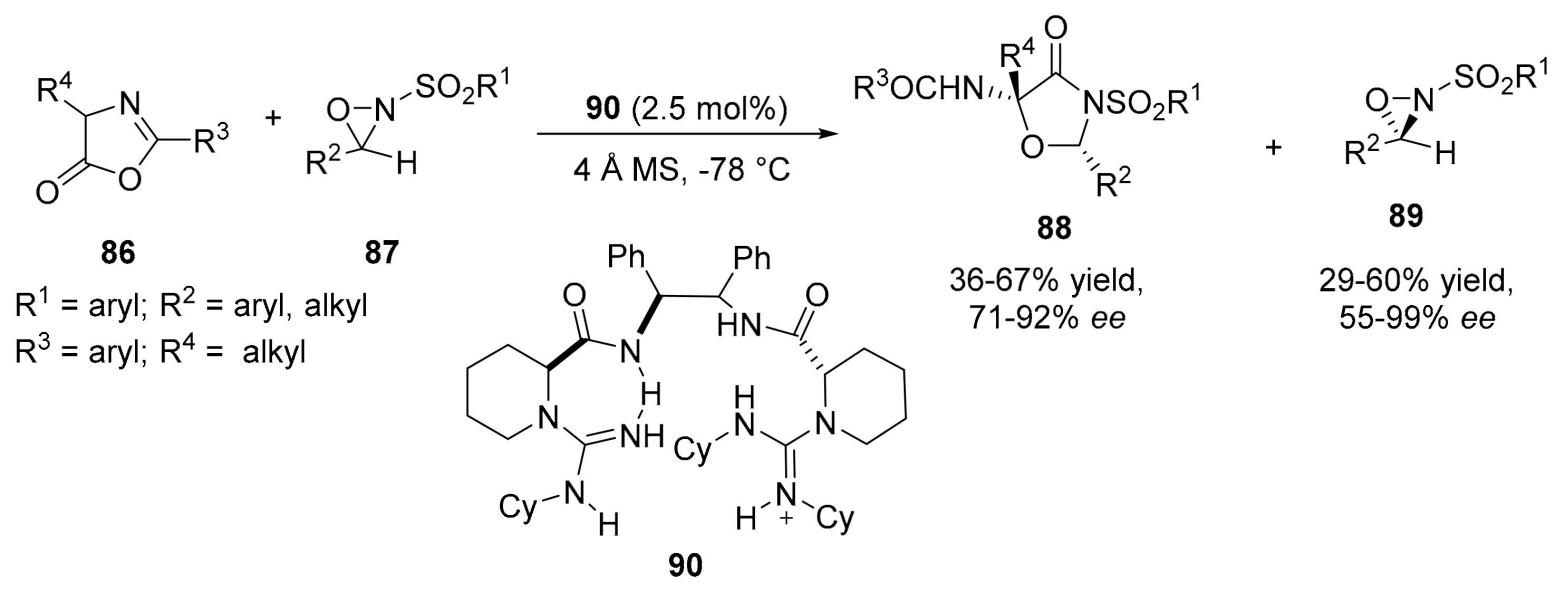
Asymmetric aminohydroxylation of azlactones with oxaziridines.

In addition to the above-mentioned alkenes and ketenes, azlactones **86** also served as the nucleophiles in the aminohydroxylations of oxaziridines **87** in the presence of a chiral bis-guanidinium salt **90** (Scheme 23) [93]. This protocol allowed the construction of a variety of optically active oxazolidin-4-ones **88** with up to 92% *ee* and the recovery of a series of enantioenriched oxaziridines **89** with good S factors.

The above-mentioned [3+2] cycloaddition aminohydroxylations of oxaziridines were based on the O-N bond cleavage. However, the study of the oxaziridine’s reactivity involving the C-O bond cleavage is much less. Troisi et al. disclosed the construction of isoxazolidines by a [3+2] cycloaddition of alkenes or alkynes with oxaziridines via the cleavage of the C-O bond [94,95]. Moreover, arynes have also been subjected to the oxaziridine to afford a set of dihydrobenzisoxazoles via the [3+2] cycloaddition [96]. Recently, the Woo group reported a visible-light photoredox-catalyzed [3+2] cycloaddition of oxaziridines via the C-O bond cleavage [97]. This methodology was a greener, atom-economical reaction of a set of oxaziridines and alkynes and afforded various 4-isoxazolines in good to excellent yield. It was noteworthy that the photoredox-catalyzed in situ generation of nitrone from the oxaziridine by single-electron transfer (SET) was involved in the reaction mechanism. 

## 5. Deracemization

**Scheme 24:**
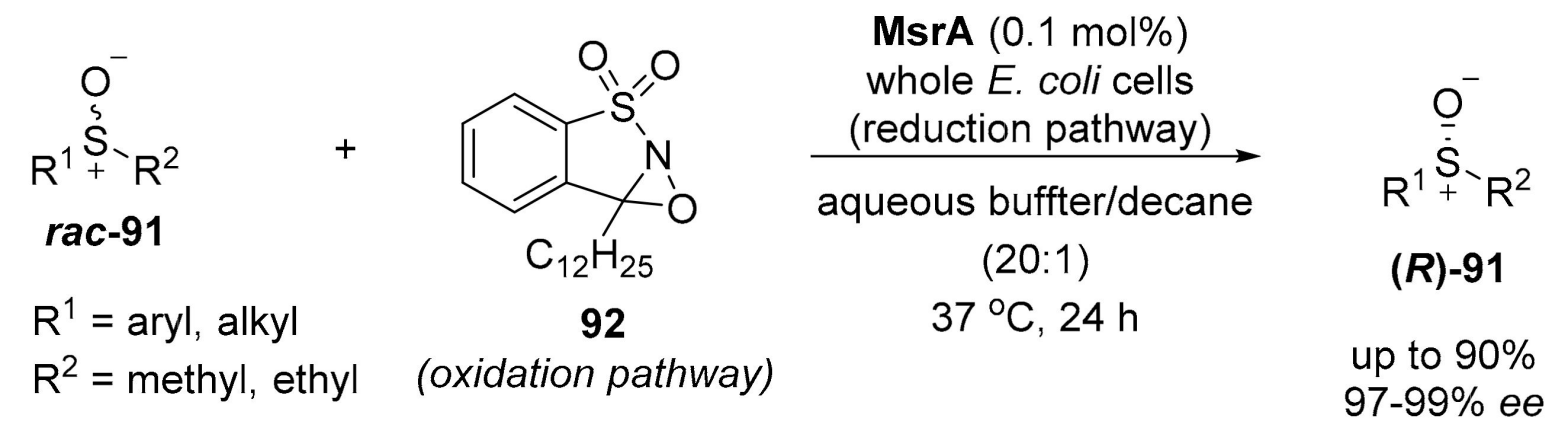
Deracemization of chiral sulfoxides.

In addition to kinetic resolution and synthesis of a chiral compound from an achiral precursor, deracemization is also an attractive and important asymmetric catalytic strategy of preparing chiral compounds. Recently, the Míšek group demonstrated the first one-pot biocatalytic deracemization of chiral sulfoxide **91** (Scheme 24) [98]. The combination of the highly enantioselective enzyme methionine sulfoxide reductase A (MsrA) and an oxaziridine-type oxidant **92** rendered high *ee* values of various aryl-alkyl and alkyl-alkyl sulfoxides. In this transformation, the lipophilic oxaziridine was utilized to oxidize the sulfide back into the racemic sulfoxide.

## 6. Conclusions

On the basis of the unusually high and tunable reactivity, the wide applications of oxaziridines have been investigated in a set of catalytic asymmetric transformations, including classic oxidation, amination and aminohydroxylation, as well as some intriguing deracemization. These protocols could efficiently and expeditiously furnish versatile and intriguing enantiopure auxiliaries and scaffolds with diverse pharmacological activities, such as optically active epoxides, sulfoxides, α-hydroxyl carbonyl compounds, α-amino carbonyl compounds, 1,3-oxazolidines and oxazolidin-4-ones. However, the catalytic asymmetric amination is still less reported. In the future, the potential synthetic utility of oxaziridines in some novel and fascinating transformations need to be further explored to achieve some important enantiopure building blocks.
